# Soil microbial communities following 20 years of fertilization and crop rotation practices in the Czech Republic

**DOI:** 10.1186/s40793-022-00406-4

**Published:** 2022-03-28

**Authors:** Martina Kracmarova, Ondrej Uhlik, Michal Strejcek, Jirina Szakova, Jindrich Cerny, Jiri Balik, Pavel Tlustos, Petr Kohout, Katerina Demnerova, Hana Stiborova

**Affiliations:** 1 Department of Biochemistry and Microbiology, Faculty of Food and Biochemical Technology, University of Chemistry and Technology, Prague, Technicka 3, 166 28 Prague 6, Czech Republic; 2 Department of Agro-Environmental Chemistry and Plant Nutrition, Faculty of Agrobiology, Food and Natural Resources, Czech University of Life Sciences Prague, Kamycka 129, 165 21 Prague – Suchdol, Czech Republic; 3 Laboratory of Environmental Microbiology, Institute of Microbiology of the CAS, Videnska 1083, 142 20 Praha 4, Czech Republic; 4 Department of Experimental Plant Biology, Faculty of Science, Charles University, Vinicna 5, 128 44 Praha 2, Czech Republic

**Keywords:** Manure, Sewage sludge, NPK fertilizers, Microbial diversity, Community structure, Chernozem, Luvisol, Cambisol

## Abstract

**Background:**

Although fertilization and crop rotation practices are commonly used worldwide in agriculture to maximize crop yields, their long-term effect on the structures of soil microorganisms is still poorly understood. This study investigated the long-term impact of fertilization and crop rotation on soil microbial diversity and the microbial community structure in four different locations with three soil types. Since 1996, manure (MF; 330 kg N/ha), sewage sludge (SF; 330 and SF3x; 990 kg N/ha), and NPK (NPK; 330 kg N/ha) fertilizers were periodically applied to the soils classified as chernozem, luvisol and cambisol, which are among the most abundant or fertile soils used for agricultural purposes in the world. In these soils, potato (*Solanum tuberosum* L.), winter wheat (*Triticum aestivum* L.), and spring barley (*Hordeum vulgare* L.) were rotated every three years.

**Results:**

Soil chemistry, which was significantly associated with location, fertilization, crop rotation, and the interaction of fertilization and location, was the dominant driver of soil microbial communities, both prokaryotic and fungal. A direct effect of long-term crop rotation and fertilization on the structure of their communities was confirmed, although there was no evidence of their influence on microbial diversity. Fungal and bacterial communities responded differently to fertilization treatments; prokaryotic communities were only significantly different from the control soil (CF) in soils treated with MF and SF3x, while fungal communities differed across all treatments. Indicator genera were identified for different treatments. These taxa were either specific for their decomposition activities or fungal plant pathogens. Sequential rotation of the three crops restricted the growth of several of the indicator plant pathogens.

**Conclusions:**

Long-term fertilization and crop rotation significantly altered microbial community structure in the soil. While fertilization affected soil microorganisms mainly through changes in nutrient profile, crop rotations lead to the attraction and repulsion of specific plant pathogens. Such changes in soil microbial communities need to be considered when planning soil management.

**Supplementary Information:**

The online version contains supplementary material available at 10.1186/s40793-022-00406-4.

## Background

The soil microorganisms represent an integral component determining the soil quality and fertility [1]. They play a pivotal role in biogeochemical cycling [2], soil health [3], plant production [4], or disease suppression efficacy [5]. The soil microbial communities are prone to changes in physical, biological, and chemical soil properties [6–8] and have a profound influence on plant development [9]. Any manipulation with these properties directly shapes the structure of microbial community and has a crucial impact on soil functioning and crop production as a consequence [10]. Therefore, the microbial community in soil reflects the impact of soil management on the overall ecosystem and should be considered when planning soil management.

Fertilization aims to maximize crop production by creating more favorable conditions for plant cultivation. The market supply includes a wide range of different kinds of fertilizers, from chemical (mineral or synthetic), biological (microbial inocultants) to organic (sewage sludge or manure), or their combinations. Chemical fertilizers are applied on the soil to address the specific needs of treated soil and cultivated crop [11]. Common chemical fertilizers aim to increase the availability primarily of nitrogen (N), and then also of phosphate (P), potassium (K), and sulphur (S). The provided nutrients are simple compounds so they can be quicky utilized by plants (e.g., ammonium nitrate, ammonium sulphate, potassium chloride, calcium nitrate, elemental sulphur, phosphate rock, superphosphate, or urea) [12]. Contrary to chemical fertilizers, organic fertilizers are complex organic materials that are slowly decomposed in soils [13]. They are commonly produced from organic wastes, such as animal manure, sewage sludge, food processing wastes, municipal solid waste, and food waste [12]. For instance, the annual production of sewage sludge is more than 10 million t_DM_ (tonnes of dry matter), and approximately 50% is used in agriculture in Europe [14, 15]. It is because recycling of such wastes fulfills the concept of “circular economy” that aims to eliminate the waste by their conversion to a valuable source of nutrients [16, 17].

The impact of fertilization on soil properties varies depending on fertilizer type and its composition [18]. Simple compounds in chemical fertilizers leads to significant increase of crop yields, but also might increase the risk of eutrophication, soil acidification, and decrease microbial biomass, biomass carbon, and microbial respiration [19, 20]. Contrary to that, the nutrients in organic fertilizers are released by microbial activity over a longer period [21]. As a result, the microbial biomass and diversity increases [22, 23] and bacterial and fungal community structures are changed [24, 25]. Furthermore, organic fertilizers augments the soil with vast numbers of microbes [26, 27]. Given the importance of microorganism in agriculture, it is especially important to monitor and evaluate the long-term effects of the fertilizers on soil microbes, and compare their long-term impact within each other.

Crop rotation is another typical agricultural practice that is used to avoid nutrient losses, enhance crop yield and mitigate the impact of soil-borne pathogens on crop production [28, 29]. Similarly to the application of fertilizer, crop rotation practices have been found to affect microbial diversity [30, 31] and microbial structures in soil [32]. The diversity and biomass have been described to increase with an increasing number of rotated crops [33–35]. Monoculture or crops growing in short rotations (two or three crops rotated on the same field) mostly lead to a decline in crop yield, which might be attributed to the enrichment of plant pathogens that can persist in the soil for the whole rotation cycle [36]. Long rotations, on the other hand, contribute to the increase of disease suppression [37]. The insertion of non-host crops into long rotations decreases the number of pathogens in the soil by not providing the host materials [36]. Unfortunately, this approach does not ensure the total elimination of soil-borne pathogens from the soil.

Our previous studies have shown how long-term fertilization influences soil enzymatic activity [38] and endophytic communities in potatoes [39]. This study focused on the effects of long-term crop rotations, and organic and chemical fertilizer application on soil bacterial and fungal communities in various soil types. The experimental fields were established at four sites in the Czech Republic that differed in their climate characteristics and soil characteristics. Chernozem, luvisol, and cambisol belong among the most widespread or the most fertile soil groups. Chernozems cover approximately 230 million ha, are among the most productive soil types in the world, and are the tenth most abundant soil group in the EU [40]. Luvisols cover approximately 600 million ha, and cambisols cover 1.500 million ha [41], being the second most abundant and the most abundant, respectively, soil groups in the EU [40]. This work aimed to: (1) determine the influence of long-term fertilization and crop rotation on microbial diversity in soils, (2) discover whether the bacterial and fungal community structures are affected, (3) describe the specific alterations of the communities if there were any, and (4) draw conclusions in the context of the soil chemistry. Since the experimental sites were established in different environments with various soil types, we expect the microbial communities to be associated primarily with these sites. However, we hypothesised that long-term fertilization regimes would influence the soil chemistry and, therefore, become the dominant driver of bacterial and fungal community structures within each experimental site. We also hypothesised that communities in organically-treated soils would differ from the chemically-treated ones because of more complex composition of manure and sewage sludge. Furthermore, the crop rotation is expected to repress specific plant pathogens and, consequently, be significantly associated with microbial community structure.

## Material and methods

### Experimental design

Four experimental field plots were established in the Czech Republic in 1996 and since then were regularly fertilized with organic and chemical fertilizers. Due to the geographically distinct locations of the field plots, different environmental conditions were achieved. Soils at the field plots differed in their soil type (determined using Casagrande’s areometric method [42]), soil chemistry, and nutrient and trace element contents (Table 1, Additional file 1: Table S1).

**Table 1:** Descriptions of experimental fields established in the Czech Republic in 1996

Location name	Hnevceves	Humpolec	Lukavec	Prague-Suchdol
GPS	50°18′46″N, 15°43′3″E	49°33′16″N, 15°21′2″E	49°33′23″N, 14°58′39″E	50°7′40″N, 14°22′33″E
Elevation (m)	265	525	610	286
Average annual temperature (°C)	8.2	7.0	7.7	9.1
Average annual rainfall (mm)	573	665	666	495
CEC (mmol_(+)_/kg)	116	90	45	262
C_ox_ (%)	0.93	1.24	1.09	1.76
pH	6.45 ± 0.5	5.27 ± 0.5	5.5 ± 0.4	7.8 ± 0.4
Bulk density (g/cm^3^)	1.50	1.40	1.27	1.43
Clay (%)	4.36	5.84	3.21	2.18
Silt (%)	76.98	43.55	37.06	71.8
Sand (%)	18.66	50.61	59.73	26.03
Soil type (WRB 2006)	Luvisol	Cambisol	Cambisol	Chernozem
NRCS USDA	Sandy loam	Silty loam	Sandy loam	Silty loam

CEC, Cation exchange capacity; NRCS, natural resources conservation service

**Fig. 1:**
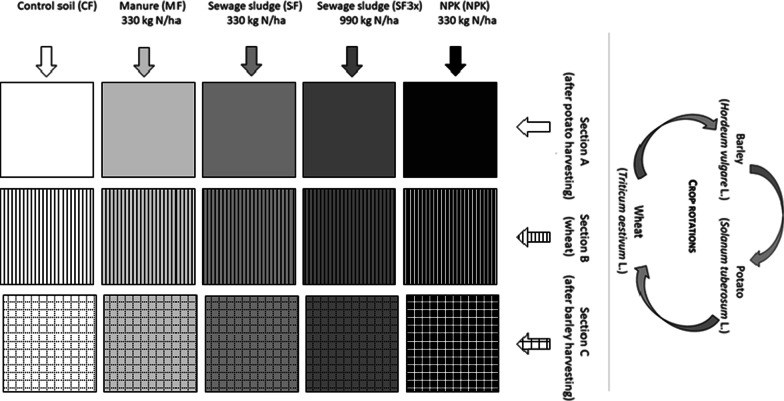
Schematic illustration of experimental design. In practice, each location had 15 sub-plots (60 m^2^) with various spatial distribution of the sub-plots. Each sub-plot also included a protection area to prevent fertiliser spreading to other sub-fileds

Experimental field plots were divided into three sections, Section A, Section B, and Section C. In each of these sections, potato (*Solanum tuberosum* L.), winter wheat (*Triticum aestivum* L.) and spring barley (*Hordeum vulgare* L.) were rotated in that order every three years, but the sections differed in a current grown crop. Another practice was the application of four different fertilizers: (1) sewage sludge (330 kg N/ha; SF), (2) sewage sludge (990 kg N/ha; SF3x), (3) cow manure (330 kg N/ha; MF), and (4) NPK (330–90–300 N-P-K kg/ha; NPK). NPK fertilizer was applied in the form of calcium ammonium nitrate (source of N), triple superphosphate (source of P) and potassium salt (source of K). Non-treated soil was used as a control (CF). Following typical agricultural practice, organic fertilizers (SF, SF3x, MF) were applied every third year in the autumn before plowing, i.e., only before potato planting. Chemical fertilizer (NPK) was applied regularly throughout the three-year rotation cycle. The application rate of fertilizers was always determined based on their nitrogen content (determined by the Kjeldahl method) for the whole three-year period. The sewage sludge used at all experimental sites always originated from a wastewater treatment plant in Prague and was stabilized anaerobically at 55 °C [43]. Anaerobic stabilization of sewage sludge took place in two stages. And the average total retention time (both stages together) was around 30 days. In contrast, different cattle manures provided by local farmers were applied to each plot. Manure originated mainly from deep cattle litter. After removal from the stable, manure was properly stored for 6–14 months in the field storage (depending on the possibilities of the experimental station). Immediately after weighing the required dose of manure at the field storage, manure is applied and plowed after application. No chemical fertilizer was applied to the organically fertilized plots. An illustration of the experimental field plots is displayed in Fig. 1. The spatial distribution of the 15 sub-plots (60 m^2^ each), each with a unique combination of fertilization treatment and crop rotation, varied at each location.

Three composite samples of bulk soil were taken from each sub-plot. Each sample consisted of six individual sub-samples distributed over a 30-cm radius collected with a soil sampler probe (0.75-in. diameter). The sub-samples were taken from the topsoil layer to a depth of 30 cm and pooled together. The total amount of soil in the composite sample, approximately 60 g, was furhter sieved through a 2 mm mesh and thoroughly mixed. In total, 180 pooled soil samples were collected from all locations in March of 2016. At the sampling time, two sections of each field plot were not vegetated: (1) Section A after potato harvesting, (2) Section C after barley harvesting. The third part, Section B, was vegetated with winter wheat. Thus, the last time the organic fertilization was applied was 0.5 years ago for Section A, 1.5 years ago for Section B, and 2.5 years ago for Section C.

For soil chemistry analyses, the three composite samples, previously sieved through a 2 mm mesh and thoroughly mixed, were compiled, further homogenized, and divided into three technical replicates. For analysis of microbial communities, an aliquot of 0.5 g of each composite sample (three representative samples per subplot) was used in downstream analyses.

### Soil chemistry analysis

Soil samples were air-dried and sieved through a 2-mm mesh. Soil pH was determined in a 0.2 mol/L KCl (2:5 w/v) solution [44]. The cation exchange capacity (CEC) was calculated as the sum of Ca, Mg, K, Na, Fe, Mn, and Al extractable in 0.1 mol/L BaCl_2_ (w/v = 1:20) for 2 h [45]. For the determination of total carbon, sulfur, and nitrogen in soils, a CHNS Vario MACRO cube (Elementar Analysensysteme GmbH, Germany) analyzer was used. In this instrument, roughly 25 mg of the soil was burned in a catalytic furnace, and C and N content was determined by using a thermal conductivity detector. Inorganic–N forms (N–NH_4_^+^ and N–NO^3−^) were determined via a SKALAR SAN PLUS SYSTEM continuous flow segmented analyzer (Skalar, Netherlands).

To determine the available macro- and micronutrient content, as well as those of potentially toxic elements in the soils, the Mehlich III extraction procedure was used. One gram of soil was mixed with 10 ml of the extraction mixture (mixed for 10 min) of the following composition: 0.2 mol/L of acetic acid, 0.25 mol/L of ammonium nitrate, 0.013 mol/L of nitric acid, 0.015 mol/L of ammonium fluoride and 0.001 mol/L of ethylenediaminetetraacetic acid (EDTA) [46]. Inductively coupled plasma-atomic emission spectrometry (ICP-OES) using an Agilent 720 (Agilent Technologies Inc., USA) equipped with a two-channel peristaltic pump, a Struman-Masters spray chamber, and a V-groove pneumatic nebulizer made of inert material was used to determine the content of Cd, Cu, Fe, Mn, Pb, Zn, P, and S in soil extracts. Spectrometry parameters were as follows: 1.2 kW; plasma flow: 15.0 L/min; auxiliary flow: 0.75 L/min; nebulizer flow: 0.9 L/min. Flame atomic absorption spectrometry (F-AAS, Varian 280FS, Varian, Australia; airflow of 13.5 L/min, acetylene flow of 2.2 L/min, burner height of 13.5 cm, and a nebulizer uptake rate of 5 mL/ min) was used to determine the content of Ca, Mg, and K in the extracts. The monitored soil chemical parameters are summarized in Additional file 1: Table S1.

### DNA isolation

Metagenomic DNA was extracted from 0.5 g of each sieved soil sample using a FastDNA™ Spin Kit for Soil (MP Biomedicals, USA) and further purified with Genomic DNA Clean and Concentrator™ (ZYMO Research, USA) according to the manufacturers’ instructions. DNA concentration and purity were determined spectrophotometrically using a NanoDrop ND-1000 (NanoDrop Technologies, USA).

### 16S rRNA gene and ITS region amplification and sequencing

Amplicons were prepared using two sequential polymerase chain reactions (PCRs) with specific primers. For 16S rRNA gene amplicons, 515 forward (5′-GTGYCAGCMGCNGCGG-3′) and 926 reverse (5′-CCGYCAATTYMTTTRAGTTT-3′) primers were used targeting the hypervariable regions V4-V5 [47]. The first 15-µl reaction consisted of 0.02 U/µl KAPA HiFi HotStart ReadyMix (Kapa Biosystems, USA), metagenomic DNA (~ 10 ng), 0.3 µM of each primer (Sigma-Aldrich, USA), and water for molecular biology (Sigma-Aldrich, USA). The temperature program for the first PCR was set as follows: 5 min at 95 °C, 20 s at 98 °C, 28—30 cycles of 15 s at 56 °C, 15 s at 72 °C, and a final extension of 5 min at 72 °C [48]. For the second PCR, which consisted of 8 – 10 cycles, the same primers as in the first PCR modified with adaptor tags and internal barcodes were used. The 25-µl reaction contained 0.02 U/µl KAPA HiFi HotStart ReadyMix (Kapa Biosystems, USA), 1 µM of each primer (Sigma-Aldrich, USA), 0.5 µl of the previous unpurified PCR product used as a DNA template, and water for molecular biology (Sigma-Aldrich, USA). The same temperature program as for the first run of PCR was used for the second PCR, except that the annealing temperature was 56 °C. The amplification of the ITS region followed the same protocol with a few modifications. Primers 5.8S-Fun forward (5′-AACTTTYRRCAAYGGATCWCT-3′) and ITS4-Fun reverse (5′-AGCCTCCGCTTATTGATATGCTTAART-3′) [49] were used and for the second PCR modified with the same adaptor tags and barcodes as the primers targeted for the 16S rRNA gene. The reactions for the first and second PCRs were prepared according to the same procedure as for 16S rRNA gene amplification. The temperature program for the first PCR was following: 5 min at 95 °C, 20 s at 98 °C, 28 – 30 cycles of 15 s at 50 °C, 15 s at 72 °C, and a final extension of 5 min at 72 °C. For the second PCR, the temperature program was the same as for 16S rRNA amplicons.

The resultant amplicons of 16S rRNA genes and ITS regions were purified using SPRIselect magnetic beads (Beckman Coulter, USA), eluted into water for molecular biology and sent on ice packs to the Core Facility for Nucleic Acid Analysis at the University of Alaska Fairbanks (AK, USA), where the sequencing analysis was performed using an Illumina Miseq platform as described earlier [48]. Briefly, the DNA concentration was normalized to 1 – 2 ng/µl using a SequalPrep Kit (Thermo Fisher Scientific, USA). With the 16S rRNA gene amplicons, the amplicons were mixed, and non-diluted, 1.5-fold diluted, and threefold diluted technical replicates were prepared and subjected to sequencing. With the ITS region amplicons, no technical replicates from sequencing were conducted, only non-diluted samples were used after the normalization of DNA concentration.

In order to maintain the proper parameters for sequence data processing, amplicons of mock communities were prepared by mixing the genomic DNA of selected strains and processed along with the amplicons of soil samples. The preparation procedure of the 16S rRNA gene mock community is described in a previous study [50], with the exception that the mock community consisted of genomic DNA of the following 12 strains: *Arthrobacter chlorophenolicus* A6, *Achromobacter xylosoxidans* A8, *Bacillus pumillus* SAFR-032, *Micrococcus luteus* NCTC 2665, *Methylobacterium radiotolerans* JCM 2831, *Pseudomonas alcaliphila* JAB1, *Pseudomonas veronii* 20a2, *Rhodobacter jostii* RHA1, *Burkholderia xenovorans* LB400, *Cupriavidus necator* H850, *Pandoraea pnomenusa* B-356, and *Rhizobium radiobacter* C58. The fungal mock community was prepared from the genomic DNA of *Saccharomyces cerevisiae* DBM 2101, *Rhodotorula mucilaginosa* DBM 19, *Fusarium culmorum* DBM 4044, *Aspergillus niger* DBM 4054, *Alternaria* sp. DBM 4245, *Candida intermedia* DSK 46, and *Penicillium chrysogenum* DBM 4062. The genomic DNA from both bacterial and fungal cultures was isolated with a DNeasy Plant Mini Kit (Qiagen, Germany), and DNA concentration was measured using a Qubit fluorimeter and Qubit® dsDNA HS Assay Kit (both Life Technologies, USA). The final mock community was prepared by mixing the genomic DNA from the cultures in equal concentrations. As a negative control 0.5 ml of water for molecular biology (Sigma-Aldrich, St Louis, MO, USA) was used and proccesed by the same manipulation as soil samples (DNA isolation and purification, PCR amplification and storage). The negative controles were done in four replicates.

### Sequence data processing

Amplicon sequence variants (ASVs) were obtained from raw sequence data using the package *DADA2* [51] in the statistical program R [52], with minor modifications to the recommended DADA2 pipeline [51] based on the results of an analysis of mock communities. Briefly, after the primers’ sequences were trimmed off, the 16S rRNA gene sequences were filtered and trimmed using the following parameters: truncLen = c(247,174), maxN = 0, maxEE = c(2,2), truncQ = 2. The ITS region sequences were filtered and trimmed using the following parameters: trimLeft = c(0, 0), maxN = 0, maxEE = c(2,2), truncQ = 10. Chimeric sequences were detected and removed using the “consensus” method. To further reduce sequencing errors, ASVs of 16S rRNA genes that differed by one base [47, 48] and ASVs of ITS region that differed by up to two bases were merged while keeping the most abundant sequence as valid. With the 16S rRNA gene sequence dataset, technical replicates from sequencing were merged while omitting all sequences that were only present in one of the replicates. The taxonomy assignment was performed with the *assignTaxonomy* function (minimal bootstrap value 50) using the rdp_train_set_16 [53] database for 16S rRNA gene sequences and the Warcup v2 database [54] for ITS region sequences. To avoid pseudoreplication, ASV abundances of samples taken from each sub-plot with a unique combination of fertilization and crop rotation were summed together. All obtained MiSeq reads were deposited in the NCBI Short Read Archive under the BioProject accession number PRJNA587449.

### Statistical analyses of soil chemistry

Highly correlated continuous chemical variables were identified using Pearson’s correlation coefficient (coefficient threshold set to |0.7|). The correlations were graphically visualized using the *psych* package [55] in R. All chemical variables were standardized by subtracting their mean value and dividing by their standard deviation. The influence of field location, fertilization, crop rotation, and interaction of these factors on soil chemistry was tested with permutational multivariate analysis of variance (PERMANOVA) based on Euclidean distance. In the final model, the variables were ordered by decreasing R^2^ values. The soil chemistry did not meet assumptions of normality (according to the Shapiro–Wilk test); hence further analyses were performed with a non-parametric Kruskal–Wallis test with a false discovery rate (*FDR*) correction of *p-*values [56]. A pairwise Wilcoxon rank-sum test was used to compare the pairs of five fertilization treatments with each other, again with an *FDR* correction of *p-*values. The same procedure was followed to test the influence of crop rotation on the soil chemistry.

### Multivariate statistical analyses of microbial community data

Further analyses of microbial data were conducted in the *phyloseq* [57] and *vegan* [58] packages in R. ASVs with no assigned taxonomy at the phylum level were removed from analyses (3.99% of all 16S rRNA gene reads; 5.67% of all ITS region reads). The analyzed datasets were rarefied to the smallest sample size (1700 for 16S rRNA ASVs and 24,000 for ITS ASVs). For testing null hypotheses, samples representing sub-plots with different combinations of fertilizer and crop rotation were permuted within the localities. Alpha-diversity was assessed by calculating Shannon and Simpson diversity indices [59]. The Kruskal Wallis rank-sum test was used to determine statistically significant differences among the diversity indices of individual treatments. The analysis of microbial diversity changes associated with different fertilization and crop rotation treatments was at first performed on the whole community dataset and then also on the datasets of each location separately.

The ASVs of prokaryotic and fungal communities were merged at the genus level and genera with ten or fewer sequences were removed. A non-metric multidimensional scaling (NMDS) based on the Bray Curtis distance was used as an ordination projection. To analyze the underlying trends affecting microbial community data, a significant correlation of environmental variables with the ordination configuration was conducted using the *envfit* function (*vegan* package). The variables that were found to significantly (*p* < 0.05) correlate with the sample distribution within the ordination space were fitted to the NMDS ordination. The length of the fitted arrows, representing the gradient direction of variables, were scaled by their correlation (square root of R^2^) so that weaker predictors had shorter arrows.

The hellinger transformation was applied to the final dataset (i.e., abundance values were divided by the total abundance, and then the square root of the result was taken) [60]. Permutational multivariate analysis of variance (PERMANOVA) was used to assess the effect of fertilization and/or crop rotation on microbial community structures. PERMANOVA was based on Bray Curtis distance, and the factors of location, soil type, fertilization, crop rotation, and their interaction were used to design the final model as follows: the test was first performed for each variable separately, then the variables were ordered according to their decreasing R^2^ values in the final model. The statistical significance of all factors was tested in this model, but the permutations never involved blending together samples from different locations. Pairwise PERMANOVA was used to identify significant differences between pairs of fertilization and crop rotation levels. Bonferroni correction [61] was used to calculate the corrected *p*-value, and permutations were again restricted within the levels of location factor. Redundancy analysis (RDA) was used to test the hypothesis that the tested factors influence hellinger-transformed microbial community data. The factors analyzed with RDA included location and soil type, fertilization regime, crop rotation, and the interaction between fertilization and crop rotation. While each of the factors was tested separately, the other factors were used as covariates to exclude their effect from the tests. An ANOVA-like permutation test was performed for RDA to assess the significance of the factors. Only those factors with *p* < 0.05 were applied in a variation partitioning analysis to assess the extent to which they explained the community data. FUNguild was used to assign identified fungal taxa to functional groups [62] followed only with plant pathogens investigation.

### Indicator genera analysis

An indicator species-like analysis (*multipatt*, *indicspecies* packages) was used to identify indicator genera for each type of treatment [63, 64]. Indicator genera are taxa that are highly associated with a specific environmental condition such as experimental treatments. The measure of association is calculated based on the probability of taxon occurrence given a sample group and probability of sample group assignment upon taxon detection [65]. The false discovery rate (*FDR*) correction method was applied to *p* values, and genera with *p*_adj_ < 0.1 were classified as the indicators. The indicator genera were confirmed to be present in a minimum of four samples from the treatment to which they were assigned.

## Results

### Chemical parameters of soils

The chemical parameters of the soils examined, including soil elements (Ca, Mg, Mn, Fe, C, Cu, S, Zn, N, Cd, Pb, P, K) and pH values, are summarized in Additional file 1: Table S1. The following positive correlations were found among the chemical parameters (Additional file 1: Fig. S1; Pearson’s correlation, coefficient threshold set to |0.7|): (1) pH with Ca and Mg, (2) Ca further correlated with C and Mn, (3) Cu with Cd and Zn, (4) N with C, (5) Fe with P. Negative correlation was observed for Fe with Mn and Ca.

The location of the experimental field plots (R^2^ = 0.54), fertilization (R^2^ = 0.27), crop rotation (R^2^ = 0.03) and the interaction of fertilization with location (R^2^ = 0.06) were all found to significantly influence the soil chemistry (*p* < 0.05, PERMANOVA). While pH significantly differed across the locations and soil types (Kruskal–Wallis test, *p*_*adj*_ < 0.05), no significant association was found between fertilization treatment and soil pH. Similarly, the levels of almost all soil elements significantly differed across the locations and soil types (Kruskal–Wallis test, *p*_*adj*_ < 0.05), and the fertilization regime was only significantly associated with 7 out of the 13 soil elements determined (Kruskal–Wallis test, *p*_*adj*_ < 0.05) (for details, see Additional file 1: Table S2). Compared to the control soil (CF), significantly higher concentrations of Cu, S, Zn, Fe, P, and N were found in SF3x treatment; Zn, Fe, P were significantly increased in SF treatment, and P with K were significantly higher in MF treatment (pairwise Wilcoxon test, *p*_*adj*_ < 0.05). In total, six elements were significantly increased in multiple treated soils, and the highest concentration of all these elements was measured in SF3x. Only one element (K) was significantly influenced by one treatment (MF), in which it reached the highest concentration in contrast to other treatments. No significant difference of soil chemistry between CF and NPK treatments was observed. Crop rotation was only significantly associated with the concentration of Cd (Kruskal–Wallis test, *p*_*adj*_ < 0.05).

### Microbial diversity

**Fig. 2:**
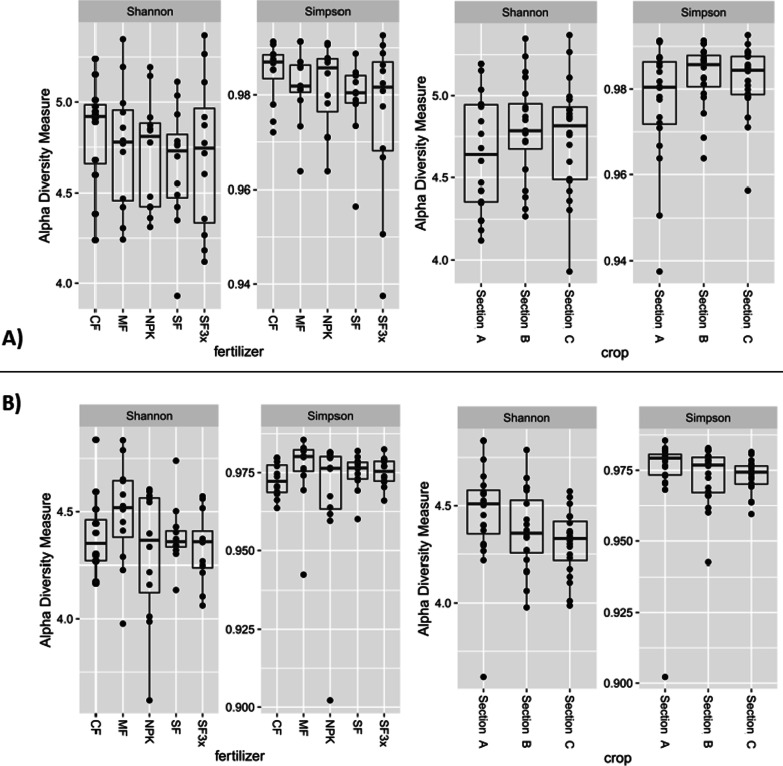
Shannon and Simpson diversity indices calculated from prokaryotic (**a**) and fungal (**b**) sequence data according to different fertilization and crop rotation treatments; (fertilizer): control (CF), cow manure (MF, 330 kg N/ha), NPK (NPK, 330–90-330 kg/ha), sewage sludge (SF, 330 kg N/ha), sewage sludge (SF3x, 990 kg N/ha); (crop): Section A (after potato), Section B (wheat), Section C (after barley)

In total, 6,405 unique 16S rRNA gene ASVs and 5,878 unique ITS ASVs were obtained from all samples. Prokaryotic and fungal Shannon and Simpson alpha-diversity indices (Fig. 2) were not significantly associated with fertilization, crop rotation or location (Kruskal–Wallis test, *p* > 0.05).

### Influence of soil chemistry on microbial communities

**Fig. 3:**
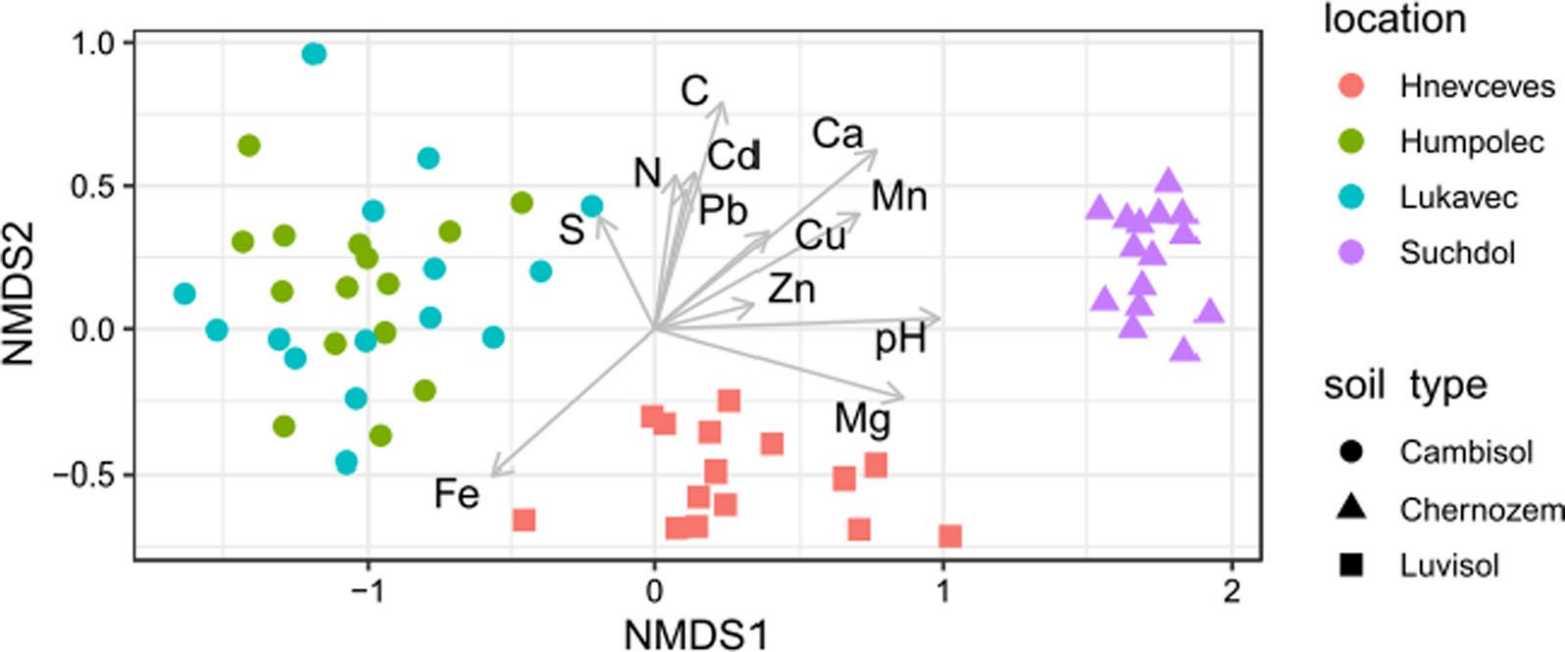
Non-metric multidimensional scaling (NMDS) ordination (stress = 0.084) of samples based on composition of soil prokaryotic community (ASVs). Samples are coded by the location (color) and soil type (symbols) they originated from. Arrows represent soil chemical parameters that significantly (*p* < 0.05) correlated with the ordination configuration

**Fig. 4:**
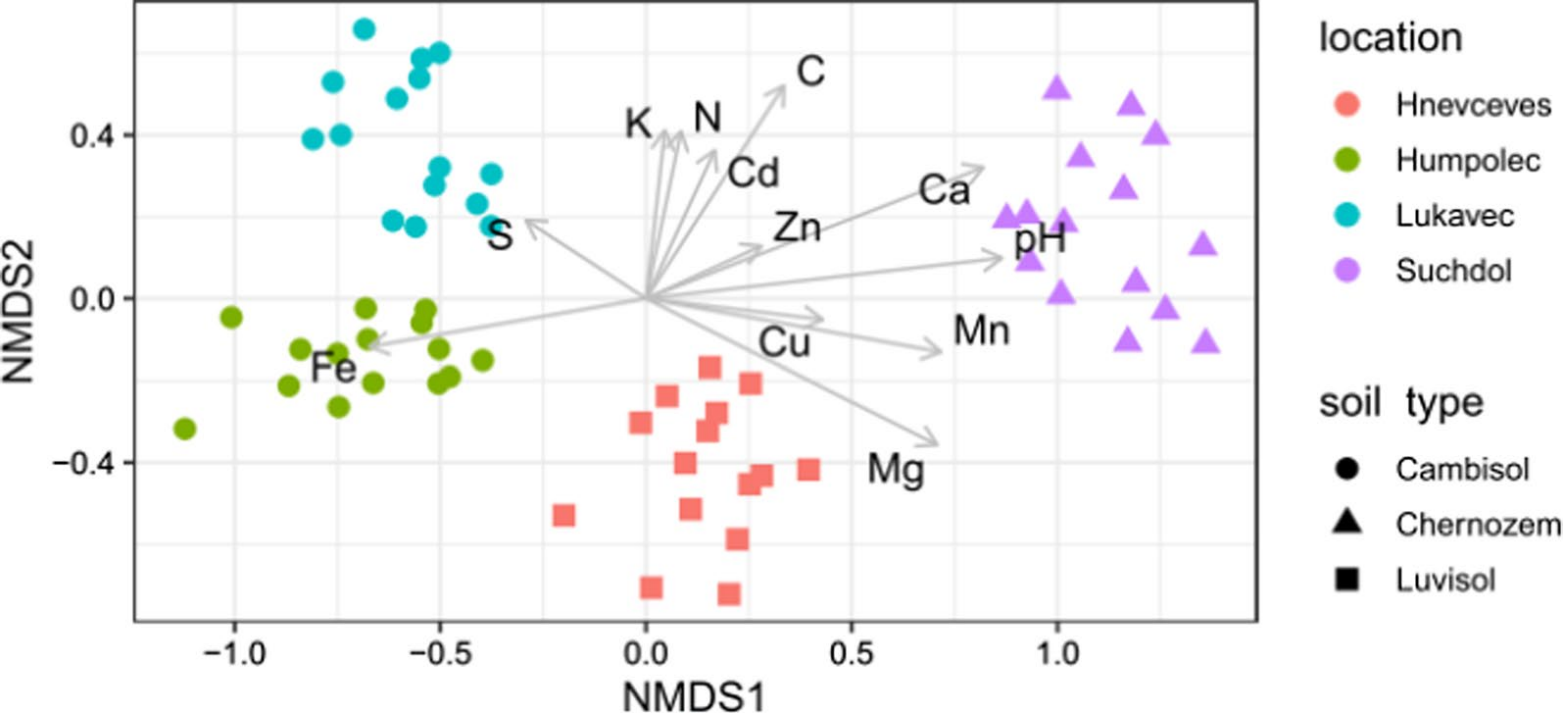
Non-metric multidimensional scaling (NMDS) ordination (stress = 0.095) of samples based on composition of soil fungal community (ASVs). Samples are coded by the location (color) and soil type (symbols) they originated from. Arrows represent soil chemical parameters that significantly (*p* < 0.05) correlated with the ordination configuration

NMDS ordinations of prokaryotic (stress 0.084, Fig. 3) and fungal communities (stress 0.095, Fig. 4) demonstrated that samples clustered according to the location and soil type. Both of these parameters were significantly correlated with the configuration of the ordination (location: R^2^ = 0.90 for both communities, *p* < 0.001; soil type: R^2^ = 0.93 and 0.87 for prokaryotes and fungi respectively, *p* < 0.001). Almost all soil chemistry parameters significantly correlated with the sample distribution as well (Figs. 3, 4). The strongest predictors for both prokaryotic and fungal communities were soil pH (R^2^ = 0.88 for prokaryotes and R^2^ = 0.85 for fungi, *p* < 0.001) and Ca (R^2^ = 0.88 for prokaryotes and R^2^ = 0.87 for fungi, *p* < 0.001). C, Fe, Mg, and Mn were other strong predictors for both prokaryotic and fungal communities (R^2^ from 0.43 to 0.72, *p* < 0.001). The only difference between predictors of the prokaryotic and fungal community was that Pb was significantly associated with prokaryotic community structure (R^2^ = 0.22, *p* < 0.001) and K only with fungal community structure (R^2^ = 0.19, *p* < 0.001). P was associated (*p* > 0.1) with neither prokaryotic nor fungal soil community structures.

### Influence of fertilization and crop rotation on microbial communities

**Fig. 5:**
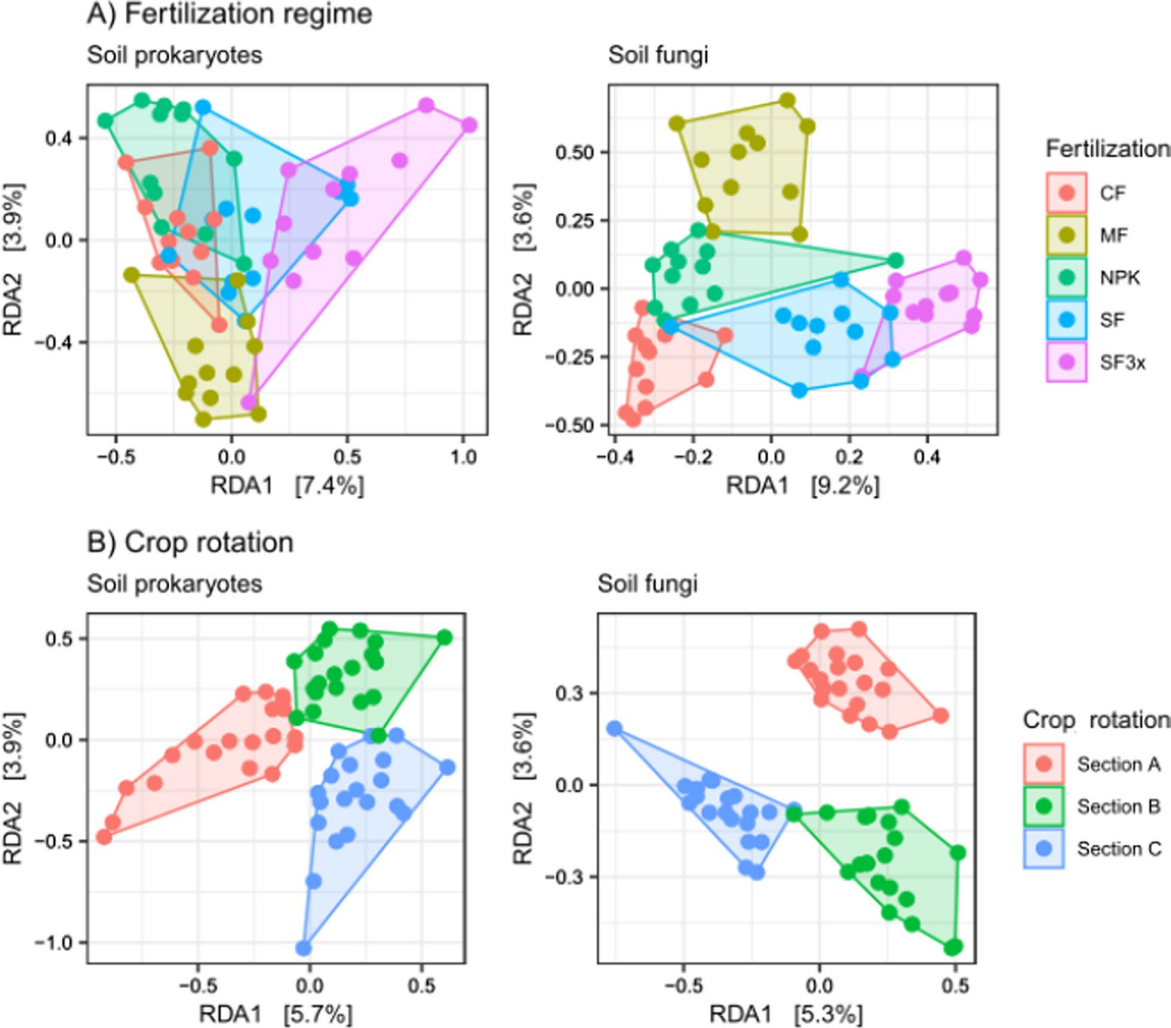
Redundancy analysis (RDA) ordinations based on Bray–Curtis distance. Subfigures represents: **a** prokaryotic and fungal community structure in soils collected from different fertilization regimes: control (CF), manure (MF; 330 kg N/ha), NPK (NPK; 330–90-330 kg/ha), sewage sludge (SF; 330 kg N/ha), sewage sludge (SF3x; 990 kg N/ha); **b** prokaryotic and fungal community structure in soils after different crop rotation: Section A (samples were collected after potato harvesting), Section B (samples were collected from field vegetated with wheat), Section C (samples were collected after barley harvesting)

The influence of fertilization and crop rotation on microbial communities was determined on clustered data of 212 prokaryotic and 402 fungal genera. Both prokaryotic and fungal community structures were significantly associated with crop rotation and fertilization regimes, after partialling out the influence of location and soil type (PERMANOVA, *p* < 0.001). The interaction effect of crop rotation and fertilization was not statistically significant on either community structure. Compared to control soil (CF), the prokaryotic community was significantly different in soils treated with SF3x and MF (Pairwise PERMANOVA, *p*_*adj*_ < 0.001), and the fungal community was significantly different (Pairwise PERMANOVA, *p*_*adj*_ < 0.05) in all fertilized soils (SF, SF3x, MF and NPK). These results correlate with RDA ordination (Fig. 5a) showing that prokaryotic communities were the most different in MF- and SF3x-treated soils from the CF soil, while fungal community structures differed in all fertilized soils. All crops included in rotations were significantly associated with both bacterial and fungal communities (Pairwise PERMANOVA, *p*_*adj*_ < 0.05) which is also shown in RDA ordination (Fig. 5b). Variation partitioning analysis showed that the influence of fertilization accounted for 5% of the variation in prokaryotes and 10% in fungi (RDA, *p* < 0.01), whereas the impact of crop rotations was 3% for both prokaryotic and fungal communities (RDA, *p* < 0.01). Location and soil type were also included in the variation partitioning analysis to determine their influence. Their significant impact (RDA, *p* < 0.001) on prokaryotic and fungal communities was 48% and 30%, respectively. Using FUNGuild, 6% of identified fungal taxa were assigned to the plant pathogen functional group. Despite location was the main driver of the structure of fungal plant pathogens in bulk soil (*R*^*2*^ = 57%, *p*_*adj*_ < 0.01, PERMANOVA), fertilizer application and crop rotation had also a significant effect on the structure (*R*^*2*^ = 4%, *p*_*adj*_ < 0.01). Specifically, a significant difference in the plant pathogen community structure was observed between CF and SF3x fertilization treatments (*p*_*adj*_ < 0.05, Pairwise PERMANOVA) and all pair comparisons of crop rotation systems (*p*_*adj*_ < 0.05).

### Analysis of indicator genera

Indicator genera associated with one or more types of fertilization regimes or crop rotation treatment are listed in Table 2. Four prokaryotic and three fungal genera were determined to be indicators of the manure amendment (MF), which included *Thermoflavimicrobium*, *Halocella*, *Clostridium* cluster XlVa (*Clostridium coccoides* group)*, Erythrobacter, Mycosymbioces, Remersonia*, and *Mycothermus*. Three prokaryotic genera, *Rhodanobacter*, *Micromonospora*, and *Candidimonas*, were found to be indicators of SF3x, whereas no indicator genera were found for SF. However, two prokaryotic (*Pseudoxanthomonas*, *Kaistia*) and two fungal genera (*Cephaliophora*, *Leucosporidium*) were found to be indicators of both SF and SF3x. One prokaryotic (*Romboutsia*) and one fungal (*Trichophyton*) genus were identified as indicators of all organic fertilization regimes (i.e., MF, SF, and SF3x). *Pseudaleuria* was found to be an indicator of all types of fertilization regimes (MF, SF, SF3x, and NPK).

**Table 2:** Indicator microorganisms for one or more types of fertilization regimes: control (CF), cow manure (MF, 330 kg N/ha), NPK (NPK, 330–90–330 kg/ha), sewage sludge (SF, 330 kg N/ha), sewage sludge (SF3x, 990 kg N/ha)

Fertilization	Kingdom	Phylum	Class	Order	Family	Genus
MF	Bacteria	Firmicutes	Bacilli	Bacillales	*Thermoactinomycetaceae_1*	*Thermoflavimicrobium*
	Bacteria	Firmicutes	Clostridia	Halanaerobiales	*Halanaerobiaceae*	*Halocella*
	Bacteria	Firmicutes	Clostridia	Clostridiales	*Lachnospiraceae*	*Clostridium_XlVa*
	Bacteria	Proteobacteria	Alphaproteobacteria	Sphingomonadales	*Erythrobacteraceae*	*Erythrobacter*
	Fungi	Ascomycota	Leotiomycetes	Helotiales	*Helotiaceae*	*Mycosymbioces*
	Fungi	Ascomycota	Sordariomycetes	Sordariales	*Sordariales_fam_Incertae_sedis*	*Remersonia*
	Fungi	Ascomycota	Sordariomycetes	Sordariales	*Chaetomiaceae*	*Mycothermus*
SF	Bacteria	Proteobacteria	Gammaproteobacteria	Xanthomonadales	*Xanthomonadaceae*	*Rhodanobacter*
	Bacteria	Actinobacteria	Actinobacteria	Actinomycetales	*Micromonosporaceae*	*Micromonospora*
	Bacteria	Proteobacteria	Betaproteobacteria	Burkholderiales	*Alcaligenaceae*	*Candidimonas*
SF, SF3x	Bacteria	Proteobacteria	Gammaproteobacteria	Xanthomonadales	*Xanthomonadaceae*	*Pseudoxanthomonas*
	Bacteria	Proteobacteria	Alphaproteobacteria	Rhizobiales	*Rhizobiaceae*	*Kaistia*
	Fungi	Ascomycota	Pezizomycetes	Pezizales	*Ascodesmidaceae*	*Cephaliophora*
	Fungi	Basidiomycota	Microbotryomycetes	Leucosporidiales	*Leucosporidiaceae*	*Leucosporidium*
MF, SF, SF3x	Bacteria	Firmicutes	Clostridia	Clostridiales	*Peptostreptococcaceae*	*Romboutsia*
	Fungi	Ascomycota	Eurotiomycetes	Onygenales	*Arthrodermataceae*	*Trichophyton*
MF, SF, SF3x, NPK	Fungi	Ascomycota	Pezizomycetes	Pezizales	*Pyronemataceae*	*Pseudaleuria*

Ten fungal genera and no prokaryotic genera were found to be indicators of different phases of three-year crop rotation (Table 3).

**Table 3:** Indicator microorganisms for different phases of crop rotation

Crop rotation	Kingdom	Phylum	Class	Order	Family	Genus
Section A (after potatoes)	Fungi	Ascomycota	Dothideomycetes	Pleosporales	*Pleosporaceae*	*Bipolaris*
	Fungi	Zoopagomycota	Zoopagomycetes	Zoopagales	*Piptocephalidaceae*	*Kuzuhaea*
	Fungi	Glomeromycota	Glomeromycetes	Glomerales	*Glomeraceae*	*Glomus*
Section B (wheat)	Fungi	Ascomycota	Leotiomycetes	Erysiphales	*Erysiphaceae*	*Blumeria*
	Fungi	Ascomycota	Dothideomycetes	Capnodiales	*Mycosphaerellaceae*	*Zymoseptoria*
	Fungi	Basidiomycota	Tremellomycetes	Filobasidiales	*Filobasidiaceae*	*Naganishia*
Section C (after barley)	Fungi	Mucoromycota	Umbelopsidomycetes	Umbelopsidales	*Umbelopsidaceae*	*Umbelopsis*
	Fungi	Ascomycota	Sordariomycetes	Magnaporthales	*Magnaporthaceae*	*Gaeumannomyces*
	Fungi	Ascomycota	Sordariomycetes	Hypocreales	*Cordycipitaceae*	*Isaria*
	Fungi	Ascomycota	Leotiomycetes	Helotiales	*Helotiaceae*	*Meliniomyces*

In each of the three sections of experimental fields, potato (*Solanum tuberosum* L.), winter wheat (*Triticum aestivum* L.) and spring barley (*Hordeum vulgare* L.) were rotated in that order every 3 years, but the sections differed in the currently grown crop: (1) Section A (samples were collected after potato harvesting), (2) Section B (samples were collected from field vegetated with wheat), (3) Section C (samples were collected after barley harvesting)

## Discussion

This study is focused on changes in soil microbial communities after 20 years of regular fertilization and crop rotation practices. The major merit of this study lies in evaluating the influence of both agricultural practices on three common soil types used for agricultural purposes and different locations. The experimental field plots were settled over 100 km apart, so large-scale gradients of environmental conditions were achieved. Temperature, cation exchange capacity, oxidizable carbon and clay content differed among the field plots (Table 1), and these factors were previously found to shape the soil microbial community [66, 67]. The soil chemistry also significantly differed across the experimental field plots, with pH being the strongest predictor of both fungal and prokaryotic community structures (Figs. 3, 4). In fact, the pH gradient was almost parallel with the first axis of NMDS ordination, reaching the highest values in chernozem soil. Soil pH was previously reported to be one of the key edaphic determinants of soil microbial community structure [68–70]. It plays, among others, a crucial role in nutrient availability for plants and the solubility of soil elements [71], and is therefore often used as a soil quality indicator [72]. Neutral soil pH, which was found in luvisol and chernozem, is mostly reported to be the optimum for nutrient availability [71].

Since location and soil type, hence soil chemistry and environmental conditions, were the major determinants of microbial community structure, they were always controlled for in further analyses when determining the influence of fertilization and crop rotation on microbial communities. Separating out the major determinants of the soil community helped us to obtain insights into the influence of agricultural practices, regardless of what other environmental factors shaped the community. Although only three soil types were included in the experiment, they are among the most widespread or fertile soils in the world [41], which makes this research globally relevant for agricultural systems.

Broadly used alpha-diversity indices, such as Shannon or Simpson, have been reported to be significantly affected after long-term fertilization [23, 73–75]. In our study, there was no evidence of a significant influence of location, soil type, long-term fertilization or crop rotation on microbial diversity, which is in agreement with other studies that found no significant effect of fertilization on either prokaryotic [76, 77] or fungal diversity [24].

However, the absence of significant changes in microbial diversity does not necessarily reflect the stability of the microbial community structure [78]. In our study, both fertilization and crop rotation were found to be significantly associated with the microbial community structures, both prokaryotic and fungal (Fig. 5). Since the soil chemistry was significantly altered by fertilization, we assume that the impact of fertilization on microbial succession was more likely indirect: first affecting soil nutrients, which subsequently affect the communities. This suggestion is in agreement with the results of other study [79]. The significant changes in soil chemistry were found in fertilized soils, especially after the application of manure (MF) or higher doses of sewage sludge (SF3x). While the manure increased concentrations of K and P, the macronutrients essential for plant growth [80], sewage sludge (SF3x) was found to be also associated with higher levels of N, P, S and heavy metals and micronutrients, such as Cu, Zn, Fe. These metals were previously detected in sewage sludge [81], and their presence raised concerns about using sewage sludge as a soil amendment [82]. It is important to note, though, that in our study, the concentration of these elements was below the risk limits [83]. Even though these heavy metals might pose a health risk at high concentrations, they are essential to plant health and growth [84].

Although the soil chemistry was significantly associated with fertilization treatments, pH was not one of the altered parameters. This finding is in contrast with our expectations and with the results of several other studies [7, 85], which showed that long-term fertilization resulted in an altered soil pH. It is important to note, though, that organic fertilizers (MF, SF, SF3x) were only applied once every three years, and the sample collection was performed at least half a year after the application of the last organic fertilizer. Therefore, we assume that temporal pH changes may occur directly upon fertilization, but did not last in the long-term. Compared to organic fertilizers, NPK application was performed periodically every year, however, this treatment was not found to significantly influence any of the monitored soil chemical parameters. Bearing that in mind, we assume that NPK doses were too low to impact the soil chemistry in the long-term.

Organic fertilizers are also a source of other substances, such as humic and fulvic acids [86] and pollutants [43, 87] when used as fertilizers. Bearing that in mind, organic fertilizers may stimulate the growth of specific indigenous populations [22] involved in the degradation of these compounds [88]. The direct influence of, in particular, organic fertilizers on soil microbial communities cannot be ruled out either; organic fertilizers contain their own microbiota [27] which are directly transferred to the soil with the application of the fertilizer [89].

Our results indicated that microbial community structure was significantly associated with the fertilization treatment, despite the fact that the final fertilizer application was performed half a year before sampling. These findings suggest that long-term fertilization has a persistent influence on both prokaryotic and fungal communities, although the impact may be different on these two communities (Fig. 5). In fact, while prokaryotic communities only significantly differed from the control soil (CF) with SF3x and MF treatments, fungal communities were significantly different from all fertilization treatments tested. Because SF3x and MF were the treatments in which the soil chemistry significantly differed the most from the control soil (CF), we conclude that the prokaryotic communities, rather than fungi, are more likely to be indirectly influenced by the fertilizers and reflect the current nutrient composition of soils. This finding is in agreement with another study [90] suggesting that nutrients, independently of other edaphic factors, strongly influence the bacterial community structure in the soil. The response of fungi, which generally have tenfold lower growth rates than bacteria [91], to soil chemistry changes is then much slower [92]. Hence, the fungal community structure does not reflect the current nutrient composition in the soil as the bacterial community.

Long-term fertilization in this study was significantly associated with the presence of specific taxa (Table 2), the majority of which are involved in nutrient cycling in soils. The fungus *Pseudaleuria*, which was found to be an indicator of all fertilization regimes, was previously associated with healthy and disease-suppressive soils [93, 94], thus emphasizing the role of fertilization regimes in the improvement of soil fitness. *Thermoflavimicrobium*, *Halocella*, and *Clostridium* cluster XIVa, all of which were found to be indicators of MF, are typical bacterial fermenters involved in the anaerobic digestion of sugars, lipids, and proteins [95], which are abundant in manure. The presence of the genus *Halocella* in manured soil was already described in other studies [96, 97], in which this genus was found to be the most dominant, together with bacilli. *Erythrobacter* was found in the later phases of the composting process of manure, suggesting that it can survive the higher temperatures that occur during compost maturation [98]. Similarly, the fungal genera *Mycosymbioces*, *Remersonia*, and *Mycothermus*, which were also found to be significantly associated with manure application, produce hemicellulosic hydrolytic and related enzymes [99, 100].

Sewage sludge was previously reported to contain several pollutants, such as polychlorinated biphenyls, polybrominated diphenyl ethers, polychlorinated dibenzodioxins, dibenzofurans, polycyclic aromatic hydrocarbons (PAHs), polyhalogenated organic compounds (hexachlorohexane, hexabromocyclododecane), heavy metals [43, 87, 101] and micropollutants, such as antibiotics [102, 103], pharmaceuticals [104] or synthetic musks [105]. These chemicals may be deposited in sewage sludge from the disposal of household chemicals, human metabolic waste, or urban runoff [106]. Nevertheless, the application of sewage sludge introduces a lot of important nutrients into arable soil, together with strains capable of degrading the pollutants [107]. In this respect, taxa commonly associated with the degradation of a wide range of organic pollutants were found to be significantly enriched in soils amended with sewage sludge. The enriched taxa included *Rhodanobacter*, which has the ability to degrade halogenated pollutants [108, 109] and high-molecular-weight PAHs [110]; *Micromonospora, Pseudoxanthomonas* and *Kaistia*, whose biodegradation roles are mostly linked with the degradation of aromatic [111] and polyaromatic hydrocarbons [112–114] as well as nitrogen-containing organic pollutants [115]; or *Micromonospora*, which is involved in the degradation of petroleum hydrocarbons [113] and other chemicals [116]. Additionaly, *Micromonospora* is also beneficial to plant health and growth. It can stimulate plant immunity by enhancing the jasmonate-regulated self-defense system [117]. Despite the fact that around 50% of sewage sludge is used as a fertilizer in Europe [14], European legislation sets the limits for metals in soil [83], while the limits for organic contaminants are not included, and the Member States have issued their own norms [14, 118]. Importantly, the concentrations of organic pollutants in our experimental soils were not significantly associated with the fertilization regime [119].

Crop rotation is an agricultural practice often used to sustain soil fertility, since the continuous planting of one crop decreases the nutrient content and promotes the build-up of host-specific plant pathogens [36]. The incorporation of other crops with different nutrient demands into the rotations helps restore the soil chemistry and decreases the number of soil-borne pathogens [36, 120]. Although microbial diversity has been reported to depend on the currently planted crop and to alter according to the order of crops rotated [30], no significant differences in diversity indices were detected in our study. As the same plants were rotated in the same order for a continuous period of 20 years, we concluded that a consistent history of long-term crop rotations contributes to the stabilization of microbial diversity. The other factor which could infuenced the unchanged diversity is the sampling the bulk soil.

Although no significant changes in alpha-microbial diversity were detected, the community structure was significantly associated with current crop rotation. Plants are able to alter the soil microbial community composition via root exudates and other rhizodeposits [8, 32, 121] which differ among plant species, cultivars or even their developmental stage [122]. Our finding indicates that the plant secondary metabolites may persist in the soil even after crop-harvesting, and together with plant residues influence the soil microbial community structure. However, further studies are needed to reveal a potential persistent influence of rhizodeposits on soil microbial communities.

A significant association between crop rotation and microbial communities was also shown by identifying indicator microorganisms that were typical of each phase of rotation. These indicators consisted only of fungi; no specific bacterial genera were found to be significantly associated with rotations (Table 3). Not surprisingly, the overwhelming majority of the observed indicator fungi were plant pathogens, except for the arbuscular mycorrhizal fungus *Glomus* [123], which was found in soil after potato harvesting. The genus *Bipolaris*, which was an indicator also found in soil after potato harvesting, includes barley and wheat hemibiotrophs [124] and is able to create dormant forms persisting in soil [125]. *Blumeria,* which was found to be associated with wheat growth, is an obligate biotrophic pathogen causing a powdery mildew of cereals, wheat included [126], and is able to produce its survival form, chasmothecia [127]. *Zymoseptoria*, detected during the wheat phase, is a biotrophic wheat pathogen [128], and *Gaeumannomyces*, detected after barley harvesting, is a soil-borne pathogen that causes the „take-all “ disease of wheat [129]. These results showed that the sequential rotation of the three crops restricted the growth of several pathogens, such as *Zymoseptoria* and *Blumeria*, over long periods, and very likely hinders outbreaks of them. However, it seems that *Bipolaris* and *Gaeumannomyces* were able to persist in soil even during the crop rotations. Since all crop rotational systems significantly shaped the profile of fungal plant pathogens in soil, it can be assumed that it is the plant secondary metabolites and plant residues that attract or repeal plant pathogens. Prolonging of the rotation cycle by the insertion of more plants might, therefore, be needed to suppress more specific plant pathogens.

## Conclusion

Our study is one of the few that investigates the effect of regular fertilization and crop rotation on soil microbial structure and diversity over the long term. The fact that the experiment was performed in multiple geographic locations with different soil types increases its general insights into how these common agricultural practices influence the soil microbiome under diverse environmental conditions. Soil chemistry that differed among the experimental filed plots was the major determinant of microbial community structures, with pH being the strongest predictor. Within the experimental field plots, the soil chemistry was further influenced by fertilization and crop rotation, which consequently modified the bacterial and fungal communities. However, the response of prokaryotic and fungal communities to long-term fertilization treatments differed. Prokaryotes significantly differed from CF only in SF3x and MF treatments while fungal communities were significantly shifted from CF by each fertilization treatment. The crop rotations significantly altered both prokaryotic and fungal communities, with the main effect on the relative abundance of plant-pathogens. In summary, our study provides deeper insights into plant-fertilizer-microbe interactions in soil, and our findings can potentially serve to improve our understanding of soil quality and globally contribute to sustainable agriculture.

## Supplementary Information

**Additional file 1:** **Table S1.** Table of soil physicochemical parameters. **Fig. S1.** Scatter plot of each pair of physicochemical parameters showing Pearson’s correlation coefficients. **Table S2.** The influence of location, soil type, fertilization and crop rotation on soil chemical properties analysed with Kruskal-Wallis test.

## Data Availability

Raw data of 16S rRNA gene amplicon sequences supporting the findings of the present study are available in the Sequence Read Archive of NCBI under BioProject accession PRJNA587449.
