# Supplementary material for: Soil microbial communities following 20 years of fertilization and crop rotation practices in the Czech Republic
Source: Environ Microbiome. 2022 Mar 28;17:13. doi: 10.1186/s40793-022-00406-4 (PMC8962459; doi:10.1186/s40793-022-00406-4)
Supplement: Supplementary file 1 — Additional file 1: Table S1. Table of soil physicochemical parameters. Fig. S1. Scatter plot of each pair of physicochemical parameters showing Pearson’s correlation coefficients. Table S2. The influence of location, soil type, fertilization and crop rotation on soil chemical properties analysed with Kruskal-Wallis test. [file 40793_2022_406_MOESM1_ESM.docx]

**Supplementary information**

**Soil microbial communities following 20 years of fertilization and crop rotation practices in the Czech Republic**

Martina Kracmarova^1🖂^, Ondrej Uhlik^1^, Michal Strejcek^1^, Jirina Szakova^2^, Jindrich Cerny^2^, Jiri Balik^2^, Pavel Tlustos^2^, Petr Kohout^3, 4^, Katerina Demnerova^1^, Hana Stiborova^🖂1^

*^1^University of Chemistry and Technology, Prague, Faculty of Food and Biochemical Technology, Department of Biochemistry and Microbiology, Technicka 3, 166 28 Prague 6, Czech Republic*

*^2^Czech University of Life Sciences Prague, Faculty of Agrobiology, Food and Natural Resources, Department of Agro-Environmental Chemistry and Plant Nutrition, Kamycka 129, Prague – Suchdol, 165 21, Czech Republic*

*^3^Laboratory of Environmental Microbiology, Institute of Microbiology of the CAS, Videnska 1083, 142 20, Praha 4, Czech Republic*

*^4^Department of Experimental Plant Biology, Faculty of Science, Charles University, Vinicna 5, 128 44 Praha 2, Czech Republic*

^🖂^**Corresponding authors:** University of Chemistry and Technology, Prague, Technicka 3, 166 28 Prague 6, Czech Republic. Phone: +420 220 44 5204; emails: [hana.stiborova@vscht.cz](mailto:hana.stiborova@vscht.cz); kracma.mk@gmail.com

**Fig. S1.** Scatter plot of each pair of physicochemical parameters showing Pearson’s correlation coefficients, histograms and bivariate scatter plots.


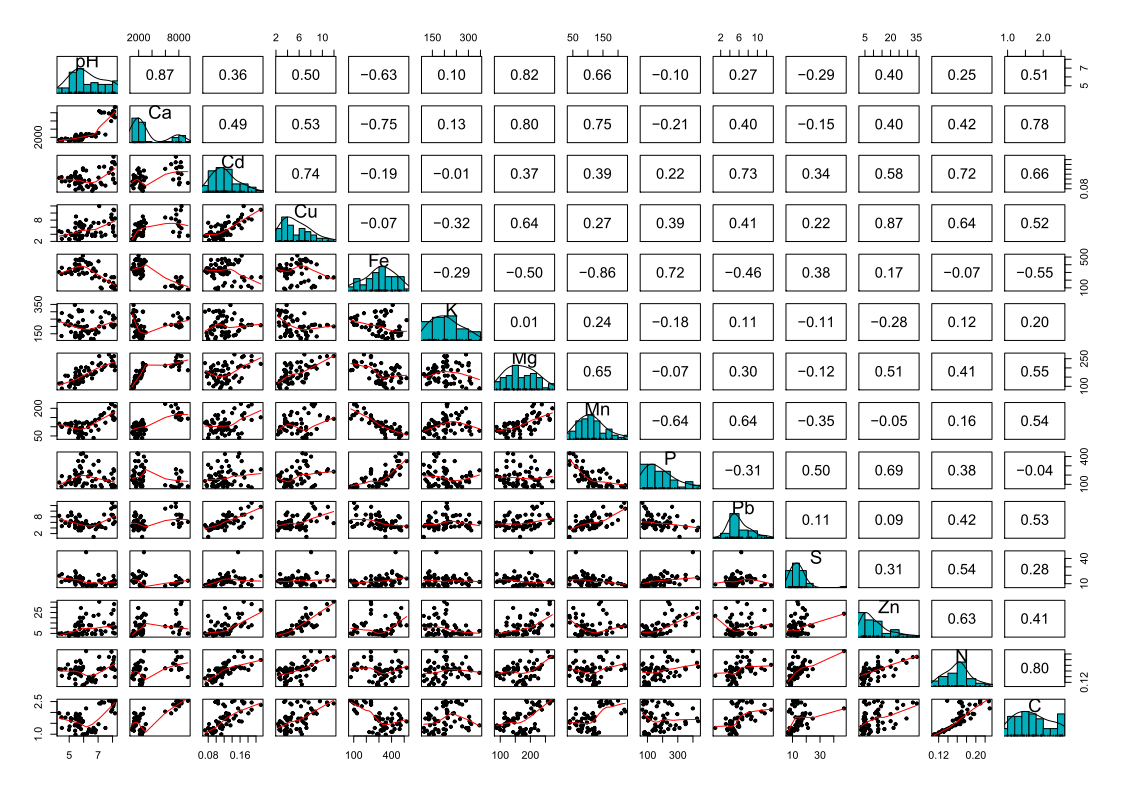


**Table S1** Physicochemical parameters of soils in the experimental fields.

| Sample ID | crop | fertilizer | location | pH | Ca [mg/kg] | Cd [mg/kg] | Cu [mg/kg] | Fe [mg/kg] | K [mg/kg] | Mg [mg/kg] | Mn [mg/kg] | P [mg/kg] | Pb [mg/kg] | S [mg/kg] | Zn [mg/kg] | N [mg/kg] | C [mg/kg] |
| --- | --- | --- | --- | --- | --- | --- | --- | --- | --- | --- | --- | --- | --- | --- | --- | --- | --- |
| HB_1 | potato | contr | Humpolec | 5.13 | 1495 | 0.132 | 3.98 | 299 | 163 | 131 | 121 | 103.5 | 9.54 | 15.5 | 4.5 | 0.1505 | 1.6235 |
| HB_2 | potato | sludge | Humpolec | 5.42 | 2068 | 0.144 | 5.86 | 405 | 138 | 147 | 81 | 212.8 | 5.62 | 24.7 | 11.82 | 0.1665 | 1.72 |
| HB_3 | potato | sludge3 | Humpolec | 6.17 | 2790 | 0.155 | 8.05 | 432 | 167 | 203 | 60 | 399.5 | 6.49 | 47.4 | 23.9 | 0.2300 | 2.1845 |
| HB_4 | potato | manure | Humpolec | 5.68 | 1660 | 0.118 | 3.68 | 274 | 303 | 184 | 123 | 123.5 | 8.74 | 16.3 | 4.39 | 0.1720 | 1.802 |
| HB_5 | potato | NPK | Humpolec | 5.17 | 1440 | 0.127 | 3.78 | 279 | 265 | 125 | 114 | 116.5 | 8.59 | 15.3 | 4.22 | 0.1590 | 1.6945 |
| HJ_1 | barley | contr | Humpolec | 5.50 | 1852 | 0.124 | 4.49 | 331 | 135 | 143 | 112 | 109.3 | 6.71 | 16.1 | 5.39 | 0.1580 | 1.683 |
| HJ_2 | barley | sludge | Humpolec | 5.73 | 2136 | 0.122 | 6.30 | 370 | 121 | 158 | 92 | 152.7 | 5.09 | 16.9 | 9.37 | 0.1640 | 1.7215 |
| HJ_3 | barley | sludge3 | Humpolec | 5.47 | 2631 | 0.130 | 9.02 | 404 | 132 | 196 | 88 | 231.4 | 6.35 | 22.8 | 12.67 | 0.1930 | 1.942 |
| HJ_4 | barley | manure | Humpolec | 4.74 | 1722 | 0.111 | 3.60 | 291 | 214 | 158 | 116 | 98.8 | 5.77 | 14.2 | 4.94 | 0.1685 | 1.7645 |
| HJ_5 | barley | NPK | Humpolec | 5.11 | 1369 | 0.116 | 4.17 | 305 | 214 | 113 | 104 | 112.9 | 6.53 | 16.4 | 4.8 | 0.1645 | 1.7385 |
| HP_1 | wheat | contr | Humpolec | 4.47 | 1625 | 0.128 | 4.61 | 279 | 135 | 117 | 101 | 75.3 | 6.71 | 12.2 | 5.05 | 0.1655 | 1.778 |
| HP_2 | wheat | sludge | Humpolec | 5.45 | 2239 | 0.149 | 7.21 | 465 | 143 | 158 | 98 | 203.7 | 7.72 | 18.6 | 13.36 | 0.1735 | 1.8535 |
| HP_3 | wheat | sludge3 | Humpolec | 5.74 | 2335 | 0.132 | 7.46 | 455 | 136 | 166 | 60 | 297 | 8.23 | 18.8 | 20.05 | 0.1965 | 1.924 |
| HP_4 | wheat | manure | Humpolec | 4.91 | 1534 | 0.125 | 4.95 | 306 | 235 | 157 | 115 | 127.7 | 8.22 | 17.5 | 5.58 | 0.1830 | 1.946 |
| HP_5 | wheat | NPK | Humpolec | 4.31 | 1335 | 0.125 | 4.86 | 304 | 240 | 115 | 117 | 120.2 | 8.35 | 19.5 | 4.85 | 0.1670 | 1.967 |
| LB_1 | potato | contr | Lukavec | 5.63 | 1370 | 0.117 | 2.50 | 335 | 227 | 113 | 79 | 157.5 | 6.04 | 12.9 | 3.07 | 0.1480 | 1.3425 |
| LB_2 | potato | sludge | Lukavec | 5.95 | 1994 | 0.164 | 5.69 | 519 | 243 | 144 | 64 | 350.5 | 4.53 | 16.1 | 12.73 | 0.1715 | 1.583 |
| LB_3 | potato | sludge3 | Lukavec | 5.96 | 1830 | 0.158 | 6.58 | 459 | 175 | 125 | 40 | 434.5 | 4.10 | 16.4 | 20.05 | 0.1750 | 1.599 |
| LB_4 | potato | manure | Lukavec | 5.71 | 1341 | 0.100 | 2.67 | 396 | 305 | 130 | 77 | 202.7 | 4.09 | 13.7 | 4.8 | 0.1690 | 1.595 |
| LB_5 | potato | NPK | Lukavec | 5.39 | 1250 | 0.136 | 3.84 | 409 | 346 | 89 | 78 | 251 | 5.94 | 12.1 | 7.4 | 0.1535 | 1.4045 |
| LJ_1 | barley | contr | Lukavec | 4.99 | 1030 | 0.093 | 2.61 | 341 | 206 | 100 | 68 | 147 | 4.79 | 13.6 | 2.75 | 0.1355 | 1.205 |
| LJ_2 | barley | sludge | Lukavec | 5.42 | 1472 | 0.105 | 3.45 | 319 | 206 | 127 | 84 | 151.3 | 4.06 | 13.7 | 6.03 | 0.1550 | 1.44 |
| LJ_3 | barley | sludge3 | Lukavec | 5.63 | 1500 | 0.104 | 6.80 | 461 | 200 | 121 | 40 | 352 | 4.12 | 14.6 | 15.4 | 0.1660 | 1.531 |
| LJ_4 | barley | manure | Lukavec | 5.65 | 1256 | 0.082 | 2.34 | 348 | 312 | 115 | 58 | 193.1 | 3.13 | 13 | 4.43 | 0.1620 | 1.467 |
| LJ_5 | barley | NPK | Lukavec | 5.03 | 1305 | 0.113 | 2.57 | 346 | 274 | 98 | 82 | 179.5 | 5.72 | 13.9 | 4.13 | 0.1485 | 1.3585 |
| LP_1 | wheat | contr | Lukavec | 5.41 | 1300 | 0.099 | 2.8 | 324 | 127 | 111 | 70 | 134 | 4.92 | 11.2 | 3.34 | 0.1305 | 1.1615 |
| LP_2 | wheat | sludge | Lukavec | 5.74 | 1605 | 0.114 | 4.76 | 472 | 140 | 121 | 62 | 287 | 4.38 | 16.8 | 12.6 | 0.1565 | 1.411 |
| LP_3 | wheat | sludge3 | Lukavec | 5.81 | 1960 | 0.122 | 7.13 | 477 | 158 | 143 | 52 | 427 | 3.12 | 17.5 | 29.35 | 0.1890 | 1.7225 |
| LP_4 | wheat | manure | Lukavec | 5.88 | 1425 | 0.107 | 2.99 | 355 | 307 | 147 | 97 | 228.5 | 5.79 | 14.4 | 5.38 | 0.1640 | 1.537 |
| LP_5 | wheat | NPK | Lukavec | 4.49 | 1095 | 0.110 | 2.73 | 361 | 291 | 88 | 99 | 188.3 | 4.64 | 21.3 | 4.58 | 0.1430 | 1.287 |
| SB_1 | potato | contr | Suchdol | 8.17 | 6864 | 0.181 | 8.48 | 126 | 211 | 207 | 211 | 80.4 | 11.6 | 9.6 | 10.95 | 0.1640 | 1.9585 |
| SB_2 | potato | sludge | Suchdol | 7.42 | 8068 | 0.171 | 7.62 | 195 | 194 | 206 | 174 | 172.3 | 8.78 | 16.4 | 15.28 | 0.1695 | 2.4565 |
| SB_3 | potato | sludge3 | Suchdol | 8.07 | 7560 | 0.212 | 10.95 | 238 | 208 | 222 | 151 | 262.5 | 11.55 | 19.1 | 27.4 | 0.1990 | 2.3485 |
| SB_4 | potato | manure | Suchdol | 8.03 | 8405 | 0.140 | 5.69 | 120 | 249 | 230 | 174 | 96.8 | 8.57 | 11.8 | 10.4 | 0.1670 | 2.4895 |
| SB_5 | potato | NPK | Suchdol | 8.16 | 9355 | 0.104 | 3.62 | 77 | 256 | 187 | 144 | 77.6 | 6.17 | 13.6 | 4.97 | 0.1385 | 2.5365 |
| SJ_1 | barley | contr | Suchdol | 7.91 | 5970 | 0.162 | 8.75 | 114 | 213 | 212 | 224 | 90.4 | 10.9 | 7.4 | 11.8 | 0.1695 | 2.0405 |
| SJ_2 | barley | sludge | Suchdol | 8.05 | 8040 | 0.122 | 6.32 | 164 | 195 | 232 | 140 | 161.5 | 5.50 | 11.7 | 14.4 | 0.1720 | 2.282 |
| SJ_3 | barley | sludge3 | Suchdol | 7.08 | 8409 | 0.169 | 11.95 | 236 | 200 | 264 | 143 | 237.2 | 5.71 | 13.7 | 35.4 | 0.2100 | 2.4125 |
| SJ_4 | barley | manure | Suchdol | 7.18 | 8131 | 0.105 | 4.48 | 104 | 262 | 225 | 163 | 92.7 | 4.60 | 13.6 | 7.55 | 0.1690 | 2.4105 |
| SJ_5 | barley | NPK | Suchdol | 7.25 | 8034 | 0.098 | 3.42 | 91 | 228 | 189 | 162 | 73.3 | 4.38 | 11.2 | 4.38 | 0.1645 | 2.4795 |
| SP_1 | wheat | contr | Suchdol | 7.98 | 7540 | 0.189 | 8.17 | 103 | 210 | 238 | 221 | 66.7 | 12.98 | 8.2 | 12.8 | 0.1820 | 2.128 |
| SP_2 | wheat | sludge | Suchdol | 7.93 | 7640 | 0.165 | 9.58 | 236 | 205 | 258 | 162 | 215.5 | 8.72 | 11.9 | 23.9 | 0.2280 | 2.5135 |
| SP_3 | wheat | sludge3 | Suchdol | 8.11 | 8295 | 0.189 | 10.8 | 238 | 211 | 264 | 137 | 246.3 | 7.70 | 14.2 | 33.07 | 0.2090 | 2.457 |
| SP_4 | wheat | manure | Suchdol | 7.75 | 7852 | 0.146 | 6.91 | 181 | 290 | 273 | 200 | 131.9 | 6.82 | 10.4 | 12.61 | 0.2095 | 2.5445 |
| SP_5 | wheat | NPK | Suchdol | 8.04 | 7165 | 0.118 | 4.69 | 129 | 274 | 212 | 181 | 72.9 | 5.46 | 9.8 | 5.84 | 0.1865 | 2.3645 |
| VB_1 | potato | contr | Hněvčeves | 6.80 | 2575 | 0.090 | 3.57 | 250 | 147 | 179 | 137 | 78.5 | 5.20 | 9.1 | 4.33 | 0.1200 | 1.079 |
| VB_2 | potato | sludge | Hněvčeves | 6.38 | 2795 | 0.115 | 5.55 | 424 | 163 | 185 | 115 | 237.2 | 4.29 | 12.3 | 12.9 | 0.1380 | 1.236 |
| VB_3 | potato | sludge3 | Hněvčeves | 7.03 | 3065 | 0.126 | 7.18 | 444 | 159 | 215 | 84 | 352 | 4.47 | 20.5 | 22.95 | 0.1665 | 1.4615 |
| VB_4 | potato | manure | Hněvčeves | 7.51 | 2665 | 0.093 | 3.86 | 267 | 300 | 214 | 122 | 147.9 | 3.94 | 7.9 | 6.9 | 0.1425 | 1.2865 |
| VB_5 | potato | NPK | Hněvčeves | 6.58 | 2290 | 0.095 | 3.91 | 223 | 176 | 143 | 120 | 82.1 | 4.78 | 6.9 | 4.55 | 0.1230 | 1.097 |
| VJ_1 | barley | contr | Hněvčeves | 5.93 | 2420 | 0.077 | 3.57 | 297 | 136 | 153 | 117 | 80.1 | 3.96 | 8.2 | 3.93 | 0.1100 | 0.9915 |
| VJ_2 | barley | sludge | Hněvčeves | 6.51 | 2385 | 0.098 | 6.25 | 408 | 116 | 165 | 78 | 209 | 4.58 | 8 | 12.6 | 0.1245 | 1.1145 |
| VJ_3 | barley | sludge3 | Hněvčeves | 6.69 | 2534 | 0.080 | 7.26 | 352 | 114 | 166 | 39 | 311.7 | 0.83 | 9.1 | 21.76 | 0.1435 | 1.2885 |
| VJ_4 | barley | manure | Hněvčeves | 6.84 | 2170 | 0.084 | 4.49 | 314 | 175 | 176 | 106 | 154.8 | 3.54 | 8.2 | 8.19 | 0.1280 | 1.1815 |
| VJ_5 | barley | NPK | Hněvčeves | 6.05 | 2200 | 0.113 | 6.46 | 284 | 196 | 184 | 118 | 110 | 5.46 | 9 | 7.17 | 0.1125 | 0.9955 |
| VP_1 | wheat | contr | Hněvčeves | 5.44 | 2483 | 0.076 | 3.79 | 354 | 156 | 175 | 128 | 91.6 | 4.27 | 8.9 | 3.49 | 0.1140 | 0.9805 |
| VP_2 | wheat | sludge | Hněvčeves | 6.31 | 2665 | 0.087 | 4.58 | 398 | 153 | 199 | 98 | 185.5 | 4.88 | 8.5 | 8.38 | 0.1310 | 1.1115 |
| VP_3 | wheat | sludge3 | Hněvčeves | 5.82 | 2824 | 0.087 | 6.12 | 464 | 197 | 203 | 80 | 278.2 | 3.44 | 10.7 | 16.42 | 0.1420 | 1.236 |
| VP_4 | wheat | manure | Hněvčeves | 6.60 | 2625 | 0.072 | 3.54 | 318 | 301 | 226 | 122 | 135 | 5.00 | 8.3 | 5.11 | 0.1340 | 1.1785 |
| VP_5 | wheat | NPK | Hněvčeves | 6.22 | 2380 | 0.091 | 3.77 | 292 | 202 | 158 | 126 | 107.5 | 5.33 | 7.1 | 4.45 | 0.1195 | 1.018 |

**Table S2** The influence of location, soil type, fertilization and crop rotation on soil chemical properties analysed with Kruskal-Wallis test. Significant *p*-values after FDR correction (*p_adj_* < 0.05) are underlined.

|  | *p_adj_* - values | | | | | | | | | | | | | |
| --- | --- | --- | --- | --- | --- | --- | --- | --- | --- | --- | --- | --- | --- | --- |
|  | pH | Ca | Cd | Cu | Fe | K | Mg | Mn | P | Pb | S | Zn | N | C |
| Location | < 0.001 | < 0.001 | < 0.001 | < 0.001 | < 0.001 | < 0.001 | < 0.001 | < 0.001 | < 0.05 | < 0.001 | < 0.001 | 0.08 | < 0.001 | < 0.001 |
| Soil type | < 0.001 | < 0.001 | < 0.001 | < 0.05 | < 0.001 | < 0.05 | < 0.001 | < 0.001 | 0.06 | < 0.001 | < 0.001 | < 0.05 | < 0.001 | < 0.001 |
| Fertilization | 0.57 | 0.25 | 0.15 | < 0.001 | < 0.05 | < 0.001 | 0.06 | 0.06 | < 0.001 | 0.48 | < 0.05 | < 0.001 | < 0.05 | 0.48 |
| Crop rotation | 0.42 | 0.85 | < 0.05 | 0.84 | 0.67 | 0.38 | 0.83 | 0.78 | 0.76 | 0.12 | 0.46 | 0.80 | 0.55 | 0.85 |
